# Long-Term Oral Administration of Salidroside Alleviates Diabetic Retinopathy in db/db Mice

**DOI:** 10.3389/fendo.2022.861452

**Published:** 2022-03-16

**Authors:** Fei Yao, Xinyi Jiang, Ling Qiu, Zixuan Peng, Wei Zheng, Lexi Ding, Xiaobo Xia

**Affiliations:** ^1^ Eye Center of Xiangya Hospital, Central South University, Changsha, China; ^2^ Hunan Key Laboratory of Ophthalmology, Changsha, China; ^3^ National Clinical Research Center for Geriatric Disorders, Xiangya Hospital, Changsha, China; ^4^ Bio-Manufacturing Engineering Laboratory, Tsinghua Shenzhen International Graduate School, Tsinghua University, Shenzhen, China

**Keywords:** salidroside, diabetes, retinopathy, oxidative stress, vascular barrier, transcriptome

## Abstract

Diabetic retinopathy (DR), a microvascular complication of diabetes mellitus, is the leading cause of vision loss in the working-age population worldwide. Unfortunately, current clinical treatments cannot completely prevent the occurrence and development of DR. Salidroside (Sal) is a medicinal supplement that has antioxidative and cytoprotective properties. This study aimed to investigate the therapeutic effect of Sal on DR. Briefly, Sal treatment was applied to wide-type mice and db/db mice (a widely used diabetic mice) at 25 mg/kg by oral gavage once daily from 8 weeks to 20 weeks. Mice’s bodyweight, blood glucose, total cholesterol, triglyceride, high density lipoprotein and low density lipoprotein were recorded and analyzed. Retinal trypsin digestion and evans blue dye assay were used to detect retinal microvessel changes and function. Retinal glutathione and malondialdehyde content measurements were applied to assess retinal oxidative stress. Full-length transcriptome analysis was performed to explore the underlying mechanisms of Sal protection. Our results found that Sal treatment could successfully relieve blood glucose and blood lipid abnormalities, and reduce retinal oxidative stress level in diabetic mice. Also, Sal treatment repaired the abnormal transcriptome caused by diabetes, alleviated the microvascular lesion of the fundus in diabetic mice, and protected retinal normal barrier function. This study enriches the indications of Sal in the treatment of diabetic diseases, providing practical research ideas for the comprehensive preventions and treatments of DR.

## Introduction

Diabetes mellitus (DM) is a group of metabolic diseases characterized by hyperglycemia, insulin resistance, and impaired insulin secretion. According to the International Diabetes Federation (IDF) statistics, the number of diabetic patients globally is around 463 million people, and it is expected to achieve 700 million in 2045 (1). As one of the most common microvascular complications of DM, diabetic retinopathy (DR) has affected 50% of type 1 diabetes and 30% of type 2 diabetes worldwide (2), and it is the leading cause of vision loss in the working-age population (3). Especially in the last 30 years, due to the aging population and the changes in lifestyles, the number of patients with type 2 diabetes has increased sharply, and the corresponding incidence rate has also shown a significantly increasing trend. The global age-standardized prevalence for blindness caused by DR has increased from 14.9% in 1990 to 18.5% in 2020 (4), indicating an urgent and severe need for DR prevention and treatment.

Retina is the only part of the body where the arteries, veins and capillaries can be directly observed with the naked eye. Retinal vascular system can effectively reflect the systemic blood circulation and microvascular changes under DM condition (5, 6). Damage to the retinal vascular system is an essential feature of the pathogenesis of DR. The typical morphological characteristics include loss of pericytes, thickening of the basement membrane, increased vascular permeability, vascular occlusion, and microaneurysms (7, 8). Retinal inflammation, oxidative stress, and glial cell dysfunction are the main pathogenic factors of DR injury (9, 10). These factors can induce damages such as destructive retinal vascular barrier, abnormal angiogenesis, and impaired retinal ganglion cell, ultimately leading to decreased vision and blindness in patients (8). So far, the clinical treatments for DR mainly include retinal laser photocoagulation, intravitreal anti-VEGF injection, sustained released dexamethasone (Ozurdex) implant, and vitreoretinal surgery (11–13). However, these treatments cannot completely prevent the occurrence and development of DR. Many patients still experience deteriorations and eventually develop blindness after clinical treatments (14). Therefore, in-depth studying the pathogenesis of DR, discovering new strategies and targets for DR treatment have crucial clinical and social significance for the prognosis of DR patients and the alleviation of medical burden (15).

Rhodiola Rosea is a precious functional medicinal plant. In China and other Asian countries, it is often used as a commercial dietary supplement to treat stress, fatigue, and altitude sickness (16–18). Salidroside (Sal) is a biologically active ingredient extracted from the Rhodiola plant, with potent antioxidant, anti-inflammatory and neuroprotective effects (19–21). This pharmacological feature can effectively target the retinal oxidative stress, retinal inflammation and nerve cell damage caused by DR. In addition, recent studies have shown that Sal can reduce the blood glucose level in diabetic mice by increasing insulin sensitivity and reducing ß-cell loss, effectively lowering diabetic deteriorations (22–24). However, the role of Sal in DR is still unclear.

In this study, we revealed that long-term application of Sal could both reduce the blood glucose levels and blood lipid levels in diabetic mice and significantly alleviate DR-induced vascular leakage and microangiopathy. Moreover, Sal treatment greatly reversed the changes in the retinal transcriptome caused by DM. All these results highlight the potential value of Sal as a dietary supplement for DR control.

## Materials and Methods

### Animals

Male wide-type C57BLKS/J mice (WT, 20-30 g, 8 weeks) and C57BLKS/J db/db mice (40-60 g, 8 weeks, genetically diabetic leptin receptor-mutated mice charactered by spontaneous obesity and hyperglycemia) were purchased from Nanjing Biomedical Research Institute of Nanjing University (Nanjing, China) and housed in a comfortable environment at 22 ± 2°C with 12 hours light/dark cycle. All the experimental procedures were approved by the Animal Ethical Committee of Xiangya hospital, Central South University (Changsha, China).

### Experimental Design

In total, 21 WT mice and 24 db/db mice were used in the study. Based on previous study (25), Sal treatment was administered at 25 mg/kg by oral gavage once daily, while the control group received water. Before the experiment, the mice were kept for 3 days to acclimate. Then WT mice and db/db mice were divided into four groups: WT group (WT mice treated with water; n = 12), WT + Sal group (WT mice treated with 25 mg/kg Sal; n = 9); db/db group (db/db mice treated with water; n = 12), db/db + Sal group (db/db mice treated with 25 mg/kg Sal; n = 12).

### Analysis of Mouse Blood Samples

Before blood samples measurement, mice were deprived of food and water for 6 hours to maintain a fasting state. Blood glucose levels in the caudal veins were measured every 4 weeks using an automatic blood glucose mete (Accu-Chek; Roche, Mannheim, Germany). Blood lipid levels, including total cholesterol (TC), triglyceride (TG), high density lipoprotein (HDL) and low density lipoprotein (LDL), were determined using an automatic blood lipid mete (CCM-111; On·call, Hangzhou, China) 12 weeks after Sal treatment. All measurements were performed and calculated according to the instructions.

### Retinal Trypsin Digestion Assay

Fresh dissected retinas were fixed in 4% paraformaldehyde for 1 hour and divided into four quadrants at the optic disk. Then retinas were digested in 3% Trypsin, 0.1 M Tris (pH 7.8) for 3 hours at 37°C until the medium became cloudy. After the incubation, the internal limiting membrane and disintegrated neuronal tissue were carefully removed, and then the remaining retinal vasculature was flattened on a glass slide for air dry. At last, air-dried retinal vasculature was treated with periodic acid solution and stained with Schiff’s reagent and hematoxylin (C0142S; Solarbio, Beijing, China). A digital imaging system was used to observe the histological change of retinal vasculature. All measurements were conducted in a masked manner and observed under the bright field.

### Evans Blue Dye Assay

Retinal vascular barrier function was determined by evans blue dye assay. Briefly, 30 mg/kg evans blue was injected through the caudal vein. Two hours later, mouse was anesthetized and perfused with 10 ml PBS to flush blood out of circulation. The retinas were collected and fixed in 4% paraformaldehyde for 1 hours. The left retina was flattened on a glass slide and observed under 652 nm excitation using a fluorescence microscope (DM5000 B; Leica, Wetzlar, Germany), the right retina was incubated with 0.3 ml formamide overnight at 70°C and centrifuged at 12,000 g for 15 minutes to extract evans blue from the retina. The absorbance of extract was determined with a spectrophotometer at 620 nm, the concentration of evans blue dye in retinal extract was calculated from the standard curve of evans blue in formamide and normalized to the dry retinal weight.

### Measurement of Retinal Oxidative Stress

Malondialdehyde (MDA) and glutathione (GSH) are crucial biochemical indicators reflecting oxidative stress levels. The retinal MDA content was measured using a Lipid Peroxidation MDA Assay Kit (S0131S; Beyotime, Shanghai, China). The retinal GSH content was measured using a Micro Reduced GSH Assay Kit (BC1175; Solarbio, Beijing, China). The measured contents were calibrated using the samples’ protein concentration.

### Full-Length Transcriptome Analysis

Mouse retinas were collected 12 weeks after Sal intervention. Two individual retinas were pooled and treated as one sample; each group contained 3 samples. TRIzol (Invitrogen, Carlsbad, CA, USA) was used to isolate total RNA, cDNA-PCR Sequencing Kit (SQK-PCS109, Oxford Nanopore Technologies Ltd, Oxford, UK) was applied to convert total RNA to cDNA. The final cDNA was then run on the PromethION platform at the Biomarker Technology Company (Beijing, China). Genes with a fold-change ≥ 1.5 identified by edgeR and a false discovery rate (FDR) < 0.05 were considered differentially expressed (BMKCloud, http://www.biocloud.net/). Gene functional annotation were based on the following databases: KEGG (Kyoto Encyclopedia of Genes and Genomes, https://www.genome.jp/kegg/) and GO (Gene Ontology, http://www.geneontology.org/).

### Statistical Analysis

SPSS version 22.0 (IBM, Armonk, NY, USA) was used for the statistical analyses. Data are expressed as the mean ± standard deviation (SD). One-way analysis of variance (ANOVA) followed by Tukey’s *post hoc* test were used for comparisons between two sets. Statistical significance was set at p < 0.05.

## Results

### Sal Improved Blood Glucose and Lipid Profiles in db/db Mice

**Figure 1:**
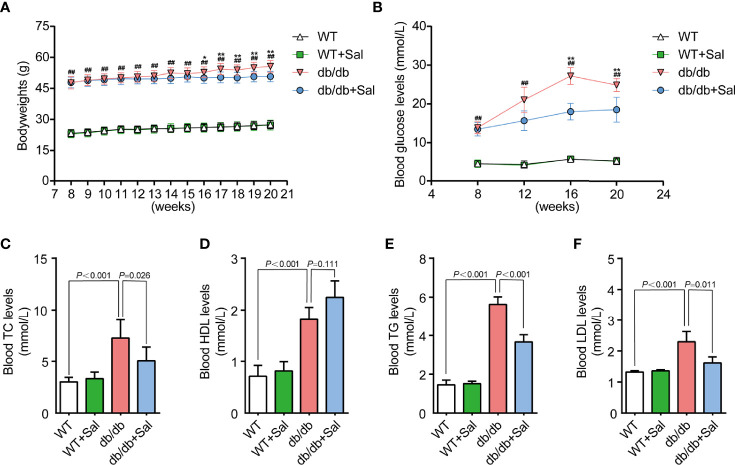
Sal improved blood glucose and lipid profiles in db/db mice. **(A)** The bodyweight of WT and db/db mice after Sal intervention. **(B)** The blood glucose of WT and db/db mice after Sal intervention. **(C-F)** The blood levels of total cholesterol (TC), high density lipoprotein (HDL), triglyceride (TG) and low density lipoprotein (LDL) in WT and db/db mice after Sal intervention. Data are the mean ± SD; ^##^p < 0.01 (db/db mice compared with WT mice), *p < 0.05, **p < 0.01 (db/db + Sal mice compared with db/db mice).

Overweight and hyperglycemia are typical features in db/db mice. As shown in **Figures 1A, B** and **Supplementary Table 1**, db/db mice showed a significant increase in bodyweight and blood glucose level compared with those in WT mice, and these pathological changes were more obvious along with aging. At week 16 and week 20, the blood glucose levels of db/db mice were nearly five times higher than that of WT mice, but such an alteration was effectively alleviated by Sal intervention. In addition, dyslipidemia is also an important feature that distinguishes db/db mice from WT mice. At 20 weeks, the contents of TC, TG, HDL and LDL in the blood of db/db mice were all greatly higher than those of WT mice. However, Sal treatment effectively improved these lipid profiles (**Figures 1C–F** and **Supplementary Table 1**). Despite Sal’s powerful function in regulating blood glucose and lipids in db/db mice, its therapeutic effect on normal WT mice was not found (**Figure 1** and **Supplementary Table 1**). Taken together, these data suggested that the long-term oral administration of Sal improved blood glucose and lipid profiles in db/db mice, indicating that the Sal is an effective and safe drug in treating dysglycemia and dyslipidemia caused by diabetes.

### Sal Alleviated Retinal Microvascular Changes in db/db Mice

**Figure 2:**
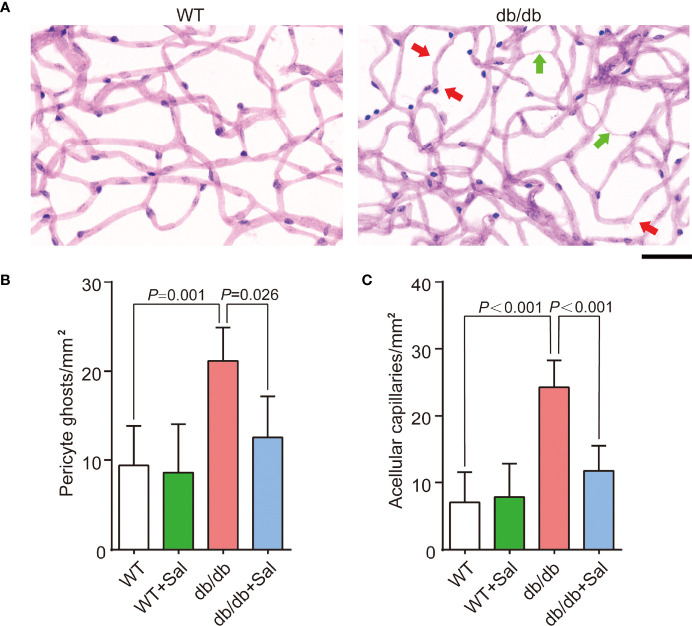
Sal alleviated retinal microvascular changes in db/db mice. **(A)** The representative photomicrographs of normal and diabetic microvessel stained by Schiff’s reagent and hematoxylin; The green arrows pointed is acellular capillaries, and the red arrows pointed is pericyte ghost. **(B)** The number of pericyte ghost in WT and db/db mice after Sal intervention. **(C)** The number of acellular capillaries in WT and db/db mice after Sal intervention. Data are the mean ± SD. Scale bar = 50 μm.

Retinal microvascular changes are the early feature DR (26). To investigate the role of Sal on DR, we quantified the number of retinal pericyte ghosts and acellular capillaries in db/db mice after Sal administration. The results showed that retinal pericyte ghosts and acellular capillaries were greatly increased in db/db mice when compared with WT mice (**Figure 2** and **Supplementary Table 1** **)**. However, Sal treatment significantly blunted the pericyte dropout and retinal acellular capillary formation in db/db mice without obvious influence on WT mice (**Figure 2** and **Supplementary Table 1**). These results demonstrated that Sal intervention alleviated retinal microvascular changes, suggesting a therapeutic effect of Sal on early DR.

### Sal Protected Retinal Vascular Barrier Function in db/db Mice

**Figure 3:**
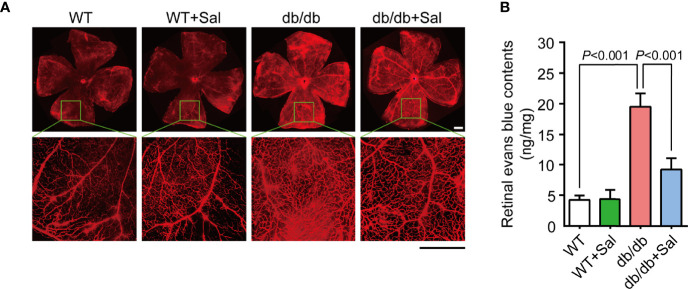
Sal protected retinal vascular barrier function in db/db mice. **(A)** The representative photomicrographs of retinal vessels stained by evans blue in WT mice and db/db mice with or without Sal intervention. **(B)** Retinal normalized evans blue contents in WT and db/db mice after Sal intervention. Data are the mean ± SD. Scale bar = 500 μm.

Pericyte loss and acellular capillaries formation could alter retinal vascular permeability and lead to vascular leakage (27). To further determine the therapeutic effect of Sal on early DR, evans blue assays were applied to assess retinal vascular barrier function in db/db mice. As shown in **Figure 3A**, db/db mice exhibited more evans blue leakage area in the retina compared with WT mice, and Sal treatment markedly alleviated this leakage extent. Evans blue quantitative results also revealed that Sal treatment greatly decreased retinal evans blue leakage levels in diabetic conditions (**Figure 3B** and **Supplementary Table 1**). All these results suggested that Sal protects retinal vascular barrier function in db/db mice, indicating a therapeutic effect on diabetes-induced retinal vascular dysfunction.

### Sal Relieved Retinal Oxidative Stress in db/db Mice

**Figure 4:**
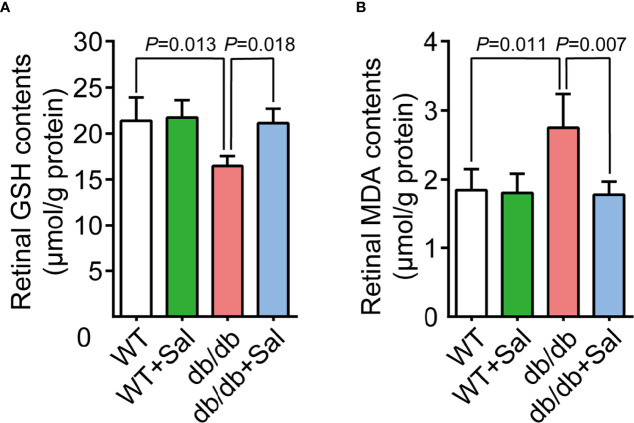
Sal relieved retinal oxidative stress in db/db mice. **(A)** Retinal GSH contents in WT and db/db mice after Sal intervention. **(B)** Retinal MDA contents in WT and db/db mice after Sal intervention. Data are the mean ± SD.

Previous studies have demonstrated that oxidative stress plays a crucial role in DR pathogenesis (28). Our results found that retinal GSH levels (an important biochemical indicator reflecting cellular anti-oxidative stress ability) were decreased in db/db mice, while Sal intervention restored retinal GSH levels in diabetic conditions (**Figures 4A** and **Supplementary Table 1**). Moreover, retinal MDA contents, a metabolite of oxidative stress, were increased in diabetic mice and were recovered to normal levels after Sal treatment (**Figures 4B** and **Supplementary Table 1**). It was noteworthy that Sal had no effect on GSH and MDA contents in WT mice. All these results suggested that Sal relieves retinal oxidative stress in db/db mice, showing an excellent antioxidant property in DR.

### Sal Ameliorated Retinal Transcriptome Abnormalities in db/db Mice

**Figure 5:**
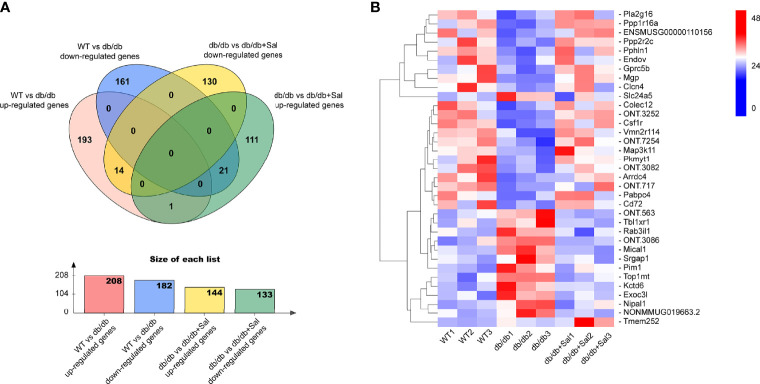
Sal ameliorates retinal transcriptome abnormalities in db/db mice. **(A)** The Venn diagram showing the differentially expressed genes (DEGs) among WT mice, db/db mice and Sal-treated db/db mice. **(B)** The heat map showing the Sal-restored DEGs (35 genes) and Sal-aggravated DEGs (1 gene) between WT and db/db mice.

To determine the underlying mechanism of Sal treatment on early DR, full-length transcriptome analysis was performed on the retina of WT, db/db and Sal-treated db/db mice at 12 weeks after intervention. As shown in **Figure 5A**, there were 208 up-regulated differentially expressed genes (DEGs) and 182 down-regulated DEGs between WT mice and db/db mice, and 144 up-regulated DEGs and 133 down-regulated DEGs between db/db mice and Sal-treated db/db mice. Among these diabetes-induced DEGs, 14 up-regulated DEGs and 21 down-regulated DEGs were completely restored by Sal treatment, and only one DEGs (*Tmem252*) was aggravated after Sal intervention (**Figures 5A, B** **)**. Except for the completely restored DEGs, Sal treatment mitigated the expression of approximately 73.08% up-regulated DEGs and 66.35% down-regulated DEGs induced by diabetes, including 8 oxidation-related genes (*Aldh4a1*, *Acad12*, *Ado*, *Kdm4d*, *Acacb*, *Mical1*, *Th* and *Htatip2*) (**Supplementary Figure 1**). These results suggested that Sal exhibits its powerful DR therapeutic effect largely depending on ameliorating retinal transcriptome abnormalities.

### The Analysis of DEGs Between db/db Mice and Sal-Treated db/db Mice

**Figure 6:**
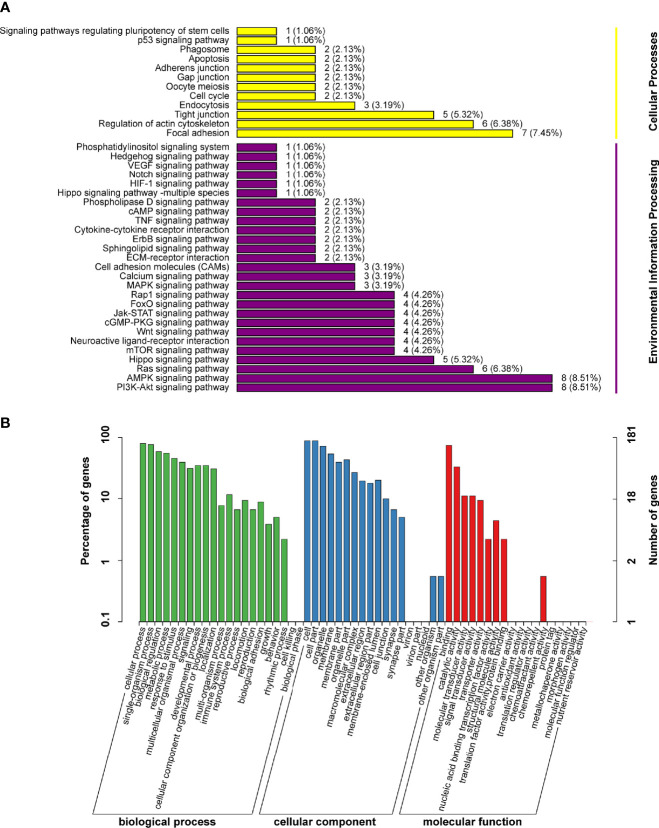
The analysis of DEGs between db/db mice and Sal-treated db/db mice. **(A)** The DEGs between db/db mice and Sal-treated db/db mice were analysed by KEGG databases. **(B)** The DEGs between db/db mice and Sal-treated db/db mice were analysed by GO databases.

To systematically identify the retinal biological process and pathways associated with Sal treatment, we performed GO and KEGG analysis on the up-regulated DEGs and down-regulated DEGs between db/db mice and Sal-treated db/db mice (**Figure 6** **).** KEGG analysis indicated that the AMPK signaling pathway and the PI3K-Akt signaling pathway were two crucial pathways after Sal treatment, as DEGs were mainly concentrated in these two pathways (**Figure 6A**).GO analysis results showed that the retinal biological process, cellular component, and molecular function were all altered by Sal intervention. The most DEGs were enriched in biological process, and the less DEGs were enriched in molecular function (**Figure 6B**).

## Discussion

DR is one of the most destructive microvascular complications of diabetes, which can lead to irreversible retinal damage (1). Although DR is not a fatal disease, it significantly influences the patient’s vision, inconveniences the patient’s daily life, and affects people’s quality of life. In addition, the cost of DR care and treatment has become an important source of medical burden for families and society. The prevention and treatment of DR has been unsatisfactory for many years. Existing clinical treatment methods have not benefited all patients, and there is yet a need to discover new therapeutic targets for DR (14, 15).

So far, DR is divided into two stages based on the degree of microvascular disease: early non-proliferative diabetic retinopathy (NPDR) and late proliferative diabetic retinopathy (PDR) (29). Patients with NPDR have no obvious symptoms in the early stage. Microaneurysm, capillary leakage and local bleeding points can be seen by fundus examination. These microvascular abnormalities increase the permeability of retinal blood vessels, causing retinal edema and the symptoms of blurred vision in patients. This stage is the best time to prevent and treat DR clinically (30). With the progression of DR, patients with NPDR may have abnormal neovascularization in the fundus while entering the PDR stage. Because of the greater fragility of neovascularization, vitreous hemorrhage is likely to be caused by easy rupture and leakage. In addition, secondary formation of retinal fibroproliferative membrane and tractional retinal detachment will induce permanent damage to patients’ vision, leading to blindness (31). At this stage, the treatment cost is higher, while the treatment outcome is far inferior to the NPDR stage (32). Therefore, early intervention, prevention and control of DR is important.

In this study, we started by giving 25 mg/kg of Sal oral treatment for diabetic mice at the early stage of abnormal blood glucose to realize the effective prevention and the control of DR through the early intervention of drugs. The results demonstrated that Sal treatment can effectively reduce the blood glucose and the blood lipid levels of diabetic mice, which is consistent with the results of some previous studies (22, 23, 25). The mechanism of its occurrence may be related to Sal improving the insulin resistance of mice and increasing the survival of pancreatic islet b cells (22). Our results also found that Sal treatment can effectively improve the fundus microvascular disease in diabetic mice, protect the blood-retinal barrier function and reduce microvascular leakage. This may benefit from regulating blood glucose and blood lipids by Sal. Many studies have shown that hyperglycemia and hyperlipidemia are substantial causes of DR through various pathways, such as protein kinase C (PKC), polyol, and hexosamine (33–35). Also, the advanced glycation end products (AGEs) induce apoptosis of vascular endothelial cells, pericytes and nerve tissues, leading to the occurrence of DR (36). Thus, the application of hypoglycemic drugs and hypolipidemic drugs can reduce the incidence of DR.

Although hyperglycemia is an important cause of DR, the impact of simply lowering blood glucose in controlling the progression of DR is limited. A large sample of clinical studies has shown that the active control of blood glucose has not significantly affected the progression of DR in patients with type 2 diabetes (37). Further, intensive blood glucose control has no remarkable benefit in improving the prognosis of DR (38). In fact, abnormal glucose and lipid metabolism in diabetic patients can interfere with the normal redox reaction in the cell, leading to an increment in ROS and oxidative stress (26). Increased ROS further induces epigenetic changes in mitochondrial enzymes, forming a metabolic memory that is unable to alleviate the symptoms of DR even when blood glucose is controlled to a normal level (39, 40). Thus, in the early stage of DR, reducing the level of oxidative stress in the retina is the key to preventing the progression of DR. As a natural antioxidant, Sal has been widely concerned about its antioxidant function (19, 41) In the retina, Sal has been proved to prevent the pigment epithelial cell damage induced by hydrogen peroxide and the vascular endothelial cell damage caused by hypoxia through reducing oxidative stress (42–45). This statement is consistent with the result of the excellent antioxidant properties of Sal in DR in this study, illustrating that the improvement of fundus microvascular disease by Sal in diabetic mice is partially due to its antioxidant activity.

The sequencing results of this study found that Sal can significantly repair the abnormal transcriptome expression caused by diabetes. Among the 390 differential genes in db/db mice and normal wild-type mice, about 70% were improved to varying degrees after the Sal treatment, 9% of which completely repaired, and only less than 0.3% of which deteriorated. This result indicated the powerful role of Sal treating DR from another perspective. Previous studies have confirmed that the P13K-AKT and AMPK signalling pathways are crucial for Sal to exert therapeutic effects by activating the above pathways to reduce diabetic-induced damages in the heart, liver, and kidney (24, 25, 46, 47). In our study, the sequencing results displayed that the retina after Sal treatment was also accompanied by the enrichment of genes related to the P13K-AKT and AMPK signalling pathways, suggesting the DR protective effect of Sal is likely to depend on the above pathways. However, the specific mechanism of action needs further studies in the future.

## Conclusion

This study explored the effects of oral Sal administration on the prevention and treatment of early diabetes and DR. Our results depicted that a long-term and low-dose oral Sal treatment can successfully relieve blood glucose and blood lipid abnormalities and reduce the oxidative stress level in the retina in diabetic mice. Also, Sal treatment repairs the abnormal transcriptome caused by diabetes, alleviates the microvascular disease of the fundus in diabetic mice, and protects the normal retinal barrier function. This study enriches the indications of Sal in the treatment of diabetic diseases, providing practical research ideas for the comprehensive preventions and treatments of DR.

## Data Availability Statement

The datasets presented in this study can be found in online repositories. The names of the repository/repositories and accession number(s) can be found below: SRA, PRJNA800617.

## Ethics Statement

The animal study was reviewed and approved by Animal Ethical Committee of Xiangya Hospital, Central South University.

## Author Contributions

FY wrote the first draft of the paper. XJ and WZ edited the paper. FY, XX, and LD designed research. FY, WZ, LQ, and ZP performed research. FY analyzed data. All authors contributed to the article and approved the submitted version.

## Funding

This work was supported by the National Natural Science Foundation of China (grant numbers 81974134 and 82171058 to XX; 82070966 to LD), the National key research and development program of China (grant numbers 2020YFC2008205 to XX), the Key R & D plan of Hunan Province of China (grant numbers 2020SK2076 to XX), the Natural Science Foundation of Hunan Province (grant numbers 2019JJ40474 to XX) and the Science and Technology Innovation Program of Hunan Province (grant numbers 2021RC3026 to LD).

## Conflict of Interest

The authors declare that the research was conducted in the absence of any commercial or financial relationships that could be construed as a potential conflict of interest.

## Publisher’s Note

All claims expressed in this article are solely those of the authors and do not necessarily represent those of their affiliated organizations, or those of the publisher, the editors and the reviewers. Any product that may be evaluated in this article, or claim that may be made by its manufacturer, is not guaranteed or endorsed by the publisher.
