# Supplementary material for: Long-Term Oral Administration of Salidroside Alleviates Diabetic Retinopathy in db/db Mice
Source: Front Endocrinol (Lausanne). 2022 Mar 16;13:861452. doi: 10.3389/fendo.2022.861452 (PMC8966089; doi:10.3389/fendo.2022.861452)
Supplement: Supplementary file 1 [file DataSheet_1.docx]

Supplementary Material

# Supplementary Figures


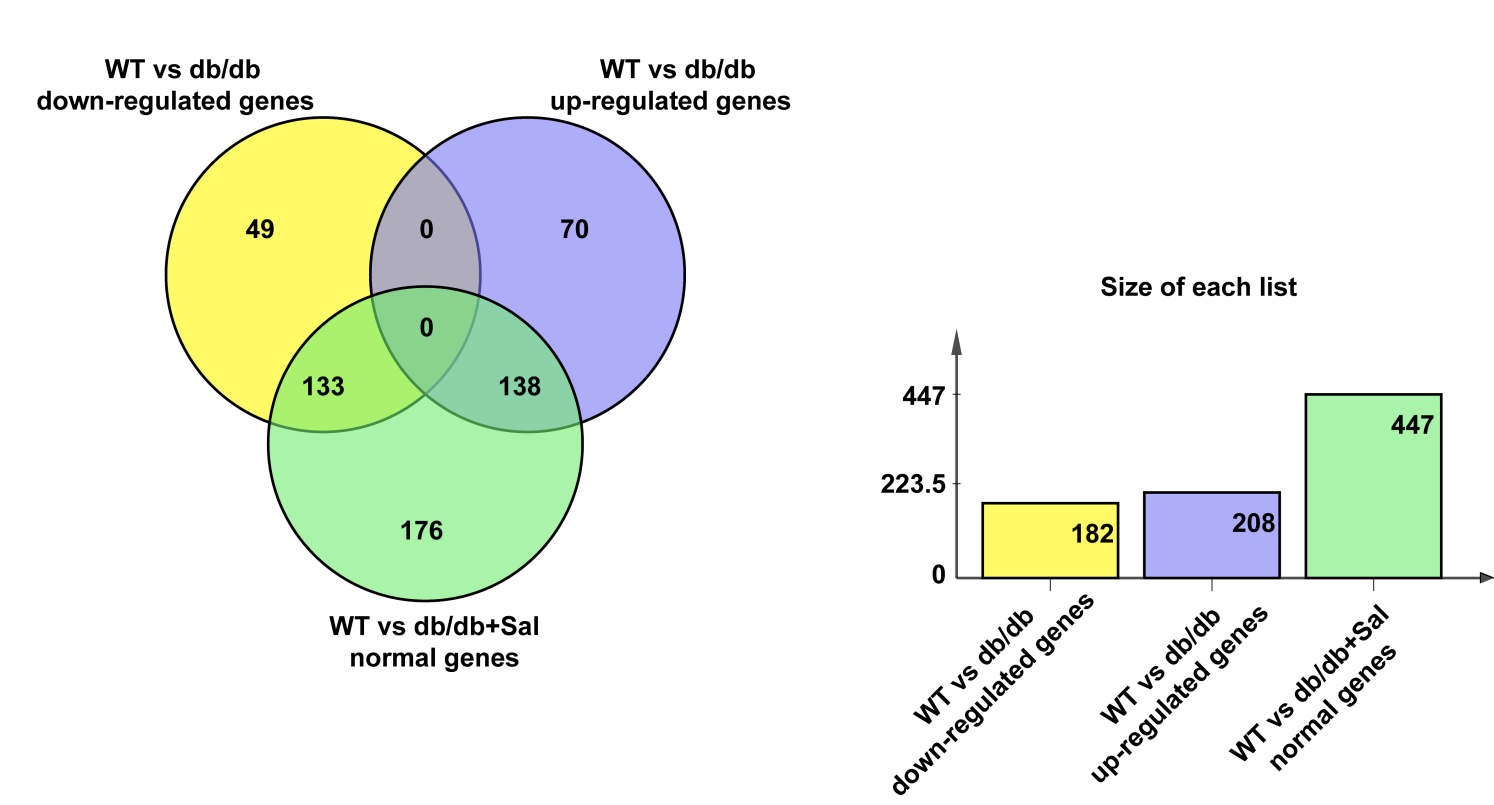


**SUPPLEMENTAL FIGURE S1 | Sal mitigated the expression of differentially expressed genes (DEGs) in retinas induced by diabetes.** db/db, db/db mice, Sal, salidroside, WT, wild type mice.

## 2. Supplementary Tables

**Supplementary Table 1**. Parameter changes in WT mice and db/db mice with or without Sal intervention.

| **Parameter**  **N= sample size** | **WT** | **WT + Sal** | **db/db** | **db/db + Sal** |
| --- | --- | --- | --- | --- |
| **Bodyweight at 8 weeks**  **(g)** | 23.34 ± 1.80  (N = 12) | 23.02 ± 1.89  (N = 9) | 47.77 ± 3.02  (N = 12) | 47.89 ± 2.59  (N = 12) |
| **Bodyweight at 9 weeks**  **(g)** | 23.81 ± 1.66  (N = 12) | 23.56 ± 1.97  (N = 9) | 49.04 ±2.66  (N = 12) | 48.79 ± 2.75  (N = 12) |
| **Bodyweight at 10 weeks**  **(g)** | 24.57 ± 1.65  (N = 12) | 24.38 ± 1.98  (N = 9) | 49.62 ± 2.50  (N = 12) | 49.24 ± 2.85  (N = 12) |
| **Bodyweight at 11 weeks**  **(g)** | 25.28 ± 1.70  (N = 12) | 25.11 ± 1.89  (N = 9) | 50.15 ± 2.47  (N = 12) | 49.69 ± 2.92  (N = 12) |
| **Bodyweight at 12 weeks**  **(g)** | 25.21 ± 1.64  (N = 12) | 24.99 ± 1.91  (N = 9) | 50.59 ± 2.54  (N = 12) | 49.62 ± 2.42  (N = 12) |
| **Bodyweight at 13 weeks**  **(g)** | 25.58 ± 1.72  (N = 12) | 25.39 ± 2.01  (N = 9) | 51.03 ± 2.62  (N = 12) | 49.63 ± 2.40  (N = 12) |
| **Bodyweight at 14 weeks**  **(g)** | 25.52 ± 1.80  (N = 12) | 25.78 ± 2.18  (N = 9) | 52.49 ± 2.69  (N = 12) | 49.93 ± 2.56  (N = 12) |
| **Bodyweight at 15 weeks**  **(g)** | 25.88 ± 1.85  (N = 12) | 25.92 ± 2.19  (N = 9) | 52.13 ± 2.78  (N = 12) | 50.67 ± 2.56  (N = 12) |
| **Bodyweight at 16 weeks**  **(g)** | 26.09 ± 1.90  (N = 12) | 26.12 ± 2.21  (N = 9) | 52.80 ± 2.65  (N = 12) | 50.09 ± 2.65  (N = 12) |
| **Bodyweight at 17 weeks**  **(g)** | 26.33 ± 1.93  (N = 12) | 26.29 ± 2.18  (N = 9) | 54.34 ± 2.68  (N = 12) | 50.36 ± 2.67  (N = 12) |
| **Bodyweight at 18 weeks**  **(g)** | 26.63 ± 2.01  (N = 12) | 26.58 ± 2.24  (N = 9) | 53.88 ± 2.55  (N = 12) | 50.18 ± 2.55  (N = 12) |
| **Bodyweight at 19 weeks**  **(g)** | 27.08 ± 2.03  (N = 12) | 26.88 ± 2.32  (N = 9) | 55.06 ± 2.63  (N = 12) | 50.72 ± 2.51  (N = 12) |
| **Bodyweight at 20 weeks**  **(g)** | 27.47 ± 2.01  (N = 12) | 27.27 ± 2.37  (N = 9) | 55.68 ± 2.90  (N = 12) | 50.75 ± 2.53  (N = 12) |
| **Blood glucose at 8 weeks (mmol/L)** | 4.60 ± 0.80  (N = 12) | 4.58 ± 0.98  (N = 9) | 13.88 ± 2.39  (N = 12) | 13.48 ± 2.74  (N = 12) |
| **Blood glucose at 12 weeks (mmol/L)** | 4.41 ± 0.91  (N = 12) | 4.20 ± 0.60  (N = 9) | 21.03 ± 5.18  (N = 12) | 15.67 ± 4.01  (N = 12) |
| **Blood glucose at 16 weeks (mmol/L)** | 5.74 ± 0.85  (N = 12) | 5.77 ± 0.85  (N = 9) | 27.23 ± 3.43  (N = 12) | 18.03 ± 3.38  (N = 12) |
| **Blood glucose at 20 weeks (mmol/L)** | 5.26 ± 0.93  (N = 12) | 5.17 ± 0.61  (N = 9) | 24.86 ± 2.64  (N = 12) | 18.48 ± 5.05  (N = 12) |
| **Blood TC**  **(mmol/L)** | 3.02 ± 0.44  (N = 8) | 3.33 ± 0.51  (N = 5) | 7.25 ± 2.13  (N = 8) | 5.06 ± 1.57  (N = 8) |
| **Blood HDL**  **(mmol/L)** | 0.71 ± 0.21  (N = 8) | 0.81 ± 0.40  (N = 5) | 1.73 ± 0.63  (N = 8) | 2.25 ± 0.38  (N = 8) |
| **Blood TG**  **(mmol/L)** | 1.46 ± 0.25  (N = 8) | 1.51 ± 0.29  (N = 5) | 5.62 ± 1.10  (N = 8) | 3.67 ± 1.06  (N = 8) |
| **Blood LDL**  **(mmol/L)** | 1.32 ± 0.04  (N = 8) | 1.36 ± 0.07  (N = 5) | 2.48 ± 0.80  (N = 8) | 1.69 ± 0.32  (N = 8) |
| **Pericyte ghosts**  **(cells/mm^2^)** | 9.40 ± 6.94  (N = 12) | 8.62 ±8.46  (N = 12) | 21.15 ± 5.84  (N = 12) | 12.53 ± 7.32  (N = 12) |
| **Acellular capillaries**  **(cells/mm^2^)** | 7.04 ± 7.08  (N = 12) | 7.83 ± 7.85  (N = 12) | 24.28 ± 6.28  (N = 12) | 11.75 ± 5.84  (N = 12) |
| **Retinal evans blue**  **(ng/mg)** | 4.25 ± 0.42  (N = 4) | 4.39 ± 0.93  (N = 4) | 19.48 ± 4.31  (N = 4) | 9.22 ± 1.85  (N = 4) |
| **Retinal GSH**  **(μmol/g protein)** | 21.38 ± 2.53  (N = 4) | 21.72 ± 1.91  (N = 4) | 16.48 ± 1.06  (N = 4) | 21.11 ± 1.58  (N = 4) |
| **Retinal MDA**  **(μmol/g protein )** | 1.85 ± 0.31  (N = 4) | 1.81 ± 0.28  (N = 4) | 2.75 ± 0.49  (N = 4) | 1.78 ± 0.19  (N = 4) |

Data are the mean ± SD unless stated otherwise.

db/db, db/db mice; GSH, glutathione; HDL, high density lipoprotein; LDL, low density lipoprotein; MDA, malondialdehyde; Sal, salidroside; TC, total cholesterol; TG, triglyceride; WT, wide-type mice.
