# Supplementary figures and images for: Long-Term Oral Administration of Salidroside Alleviates Diabetic Retinopathy in db/db Mice
Source: Front Endocrinol (Lausanne). 2022 Mar 16;13:861452. doi: 10.3389/fendo.2022.861452 (PMC8966089; doi:10.3389/fendo.2022.861452)

Raw data download links：

<https://www.jianguoyun.com/p/DX5P-XMQpNCcChjmpKoE>


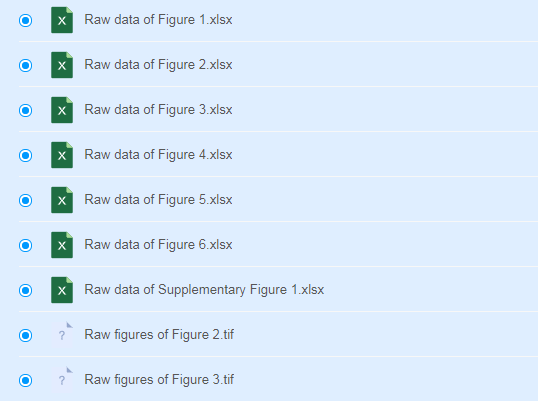

Supplement: Supplementary file 2 [file DataSheet_2.docx]
